# Pig-Posture Recognition Based on Computer Vision: Dataset and Exploration

**DOI:** 10.3390/ani11051295

**Published:** 2021-04-30

**Authors:** Hongmin Shao, Jingyu Pu, Jiong Mu

**Affiliations:** 1 College of Information Engineering, Sichuan Agricultural University, Ya’an 625000, China; shaohongmin@stu.sicau.edu.cn (H.S.); pujingyu@stu.sicau.edu.cn (J.P.); 2 Sichuan Key Laboratory of Agricultural Information Engineering, Ya’an 625000, China

**Keywords:** computer vision, posture recognition, pig posture, agricultural automation, automated breeding

## Abstract

**Simple Summary:**

This study explored an image-based method for recognizing pigs’ postures during growth and established the world’s first human-annotated pig-posture-recognition dataset, which includes pigs standing, lying, lying on their sides, and exploring (the four common postures). Finally, the pig postures were judged, and good results were obtained in practical applications.

**Abstract:**

Posture changes in pigs during growth are often precursors of disease. Monitoring pigs’ behavioral activities can allow us to detect pathological changes in pigs earlier and identify the factors threatening the health of pigs in advance. Pigs tend to be farmed on a large scale, and manual observation by keepers is time consuming and laborious. Therefore, the use of computers to monitor the growth processes of pigs in real time, and to recognize the duration and frequency of pigs’ postural changes over time, can prevent outbreaks of porcine diseases. The contributions of this article are as follows: (1) The first human-annotated pig-posture-identification dataset in the world was established, including 800 pictures of each of the four pig postures: standing, lying on the stomach, lying on the side, and exploring. (2) When using a deep separable convolutional network to classify pig postures, the accuracy was 92.45%. The results show that the method proposed in this paper achieves adequate pig-posture recognition in a piggery environment and may be suitable for livestock farm applications.

## 1. Introduction

According to the National Bureau of Statistics of China, China is a major consumer of pork. A total of 544.19 million live pigs were sold in 2019 in China, accounting for 45.08% of the total sold globally and ranking China first in the world for pig sales [1]. In 2017, the output value of pig feeding in China was nearly 1.3 trillion yuan, accounting for about 56.5% of the total output value of domestic livestock and poultry (pigs, cattle, sheep and poultry) [2]. With an increase in the scale of the pig breeding industry, the possibility of swine diseases also increases. A failure to intervene or eliminate these hidden dangers over time may cause serious economic and human losses. For example, African Swine Fever (ASF), which has had a huge impact on the world in recent years, is a virulent, infectious swine disease caused by the ASF virus, which is clinically characterized by acute febrile hemorrhage, high morbidity, and high mortality [3]. It poses a serious danger to the development of the pig industry. An epidemic not only incurs great economic losses for the affected country but also hampers international trade. Many diseases that may occur in the growth of live pigs are accompanied by changes in their postures before other subclinical and clinical symptoms emerge, which, at the same time, lead to changes in various external physiological parameters, such as decreased daily activities, a loss of appetite, and changes in their cries. Monitoring pigs’ posture can reveal the precursors of swine disease in a timely manner, identify the factors threatening the health of pigs in advance, detect and control the source of infection, and reduce the scope of infection, which will help to slow down the transmission of severe infectious diseases in the pig population [4].

Secondly, the traditional pig industry requires breeders to obtain real-time information about the statuses of pigs so as to detect abnormalities, but the traditional observation method is time consuming and laborious, and interferes with the normal growth of pigs. At present, some breeders observe the statuses of pigs by watching surveillance videos, but this is time consuming, and, in most cases, breeders cannot accurately identify the postures of pigs.

In recent years, technology led production systems have developed rapidly. For example, radio frequency identification (RFID) technology is now being used to identify the individual characteristics of pigs [5], and microinertial sensors are being used to monitor pig posture [6]. Various sensors, such as those for pressure, water volume, and acceleration, are also used to monitor lameness, drinking water, movement, and other postures [7,8,9]. However, these methods all rely on wearable hardware. In practical applications, sensors used at close range in denser piggeries are prone to damage or falling off.

With the development of deep-learning technology, image processing has been gradually enhanced. It is mostly applied for animal-posture recognition in animal husbandry [10]. For example, Leroy et al. automatically recognized the standing, walking, and scratching postures of a single laying hen through a distribution model that used the elliptical fitting of points to measure the contours of laying hens [11]. Jiazhen Han used the Densenet model to realize the multi-label classification of dairy goat datasets [12]. However, most of these methods are based on individual identification, while in pig breeding, group breeding is the main method used and the pigs are generally clustered close together. L Zhang et al. used key point tracking, triangulation, and other methods to detect multiple pigs. This type of method solves the problem of occlusion between pigs. In most production environments, background factors in the collected images greatly impact the recognition effect, and this effect is not ideal after image marginalization processing.

Object detection is an important research direction in computer vision. Its purpose is to accurately identify the category and location of a specific target object in a given image. In recent years, the feature learning and transfer learning capabilities of deep convolutional neural networks (DCNN) have significantly progressed in the feature extraction, image expression, classification, and recognition for object-detection algorithms [13]. Image-segmentation technology is a process that involves dividing an image into several regions with different characteristics based on the principle of similarity, while semantic image segmentation is based on common pixel points and processes images at the pixel level [14]. These two methods have also been applied in many agricultural fields in recent years. For example, Huimin Liao used the RGB-D (RGB+ Depth Map) method to segment individual images from groups [15], while Xiangze Lin used Mask R-CNN to quickly locate and identify rice planthoppers [16]. Though the target-detection method has a high recognition speed, it cannot remove the influence of the background. However, the semantic-segmentation method can segment the target completely.

In order to make pig-posture recognition more accurate, this study established the world’s first human-annotated pig-posture-recognition dataset, including four pig postures: standing, lying on the stomach, lying on the side, and exploring. All the experiments described in this paper were based on this dataset.

**Figure 1:**
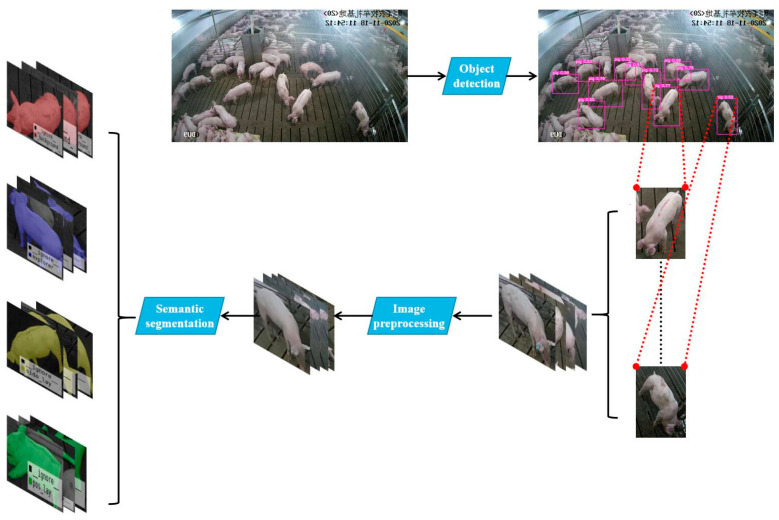
The overall framework of the envisaged method. First, we extracted a single target from the original dataset of multiple targets; then, we preprocessed the image of the single target, extracted the action features and, finally, carried out semantic segmentation and classification.

To solve the abovementioned problems, this paper proposes a hybrid model that combines object detection and semantic segmentation. First, we used the YOLOv5 object-detection method to extract individual pigs from pig-group pictures, and then, we used the DeepLab v3+ semantic-segmentation method to extract the individual contours of the pigs. The experimental results show that the method proposed in this paper adequately recognizes the postures of pigs in a piggery environment. The overall framework of the method is shown in Figure 1.

This paper is divided into five sections. This section introduces the significance and objectives of pig-posture research. The second section describes the network principles used and compared in this paper. The third section specifically introduces the dataset and explains the image-preprocessing method. The fourth section describes the training process and experimental results for target extraction and classification, and analyzes the results obtained with different processing methods. The fifth section summarizes the findings and makes suggestions for future research.

## 2. Related Work

### 2.1. YOLOv5-Based Detection Network for a Single Pig

Object detection is a form of image segmentation based on the geometric and statistical features of a target that also combines the segmentation and recognition of the target. The current deep-learning methods in the field of object detection are mainly divided into two categories. The one-stage target-detection algorithm is an end-to-end process, no candidate frames are required, and the positioning problem of the target frame is directly transformed via regression-problem processing, such as YOLO [17] or SSD [18]. The two-stage object-detection algorithm is based on the candidate area: the algorithm first generates a series of candidate frames as samples and then classifies the samples through the convolutional neural network. In the early stage of the development of end-to-end algorithms, such as YOLOv1, speed is the main advantage, while methods based on the candidate region have an advantage in terms of detection accuracy and precision. However, YOLOv1 has the following two shortcomings: (1) Due to the output layer for the fully connected layer, in the test, the YOLO training model only supports and trains at the same input-image resolution. (2) Although each grid can predict the B bounding boxes, only the bounding box with the highest intersection over union (IoU) is selected as the object detection output; that is, each grid can only predict one object at most. When objects occupy a small proportion of the picture, such as when the image contains a flock of birds, each grid contains multiple objects, but only one of them can be detected [17]. YOLOv2 proposed a new training method: the joint training method. The basic idea is to train the object detector on both the detection and classification datasets at the same time, to learn the exact position of the object with the data from the detection dataset, and to increase the number of categories and improve the robustness of the data from the classification dataset [19]. YOLOv3 introduces multi-scale prediction, and independently performs detection on the fusion feature maps of multiple scales, ultimately improving the detection effect of small targets significantly [20]. YOLOV4 [21] and YOLOV5 have a greatly improved training speed and accuracy, so this study adopted YOLOV5 as the first step of the joint training method.

### 2.2. DeepLabv3+-Based Edge-Extraction Network for a Single Pig

Image segmentation is another basic task in computer vision, in addition to classification and detection. It involves segmenting the picture into different blocks according to the content. DeepLab is a semantic-segmentation model proposed by Google. It improves the feature extractor of the convolutional neural network (CNN), which can model objects more successfully and interpret contextual information more accurately [22]. DeepLabv3+ is the latest improvement to the DeepLab model [23]. The main body of the DeepLabv3+ Encoder is DCNN with dilated convolutions. Common classification networks such as Resnet and Xception can be used, followed by atrous spatial pyramid pooling (ASPP) with dilated convolutions, to control the receptive field without changing the size of the feature map, which is beneficial for the extraction of multi-scale information. Compared with DeepLabv3, the direct bilinear interpolation to the original image size after the 1 × 1 classification layer is not conducive to obtaining finer segmentation results [24]. V3 + introduces a new DECODER module to further integrate low-level features with high-level features in order to improve the accuracy of the segmentation boundaries.

### 2.3. Feature-Extraction Network

#### 2.3.1. Resnet

**Figure 2:**
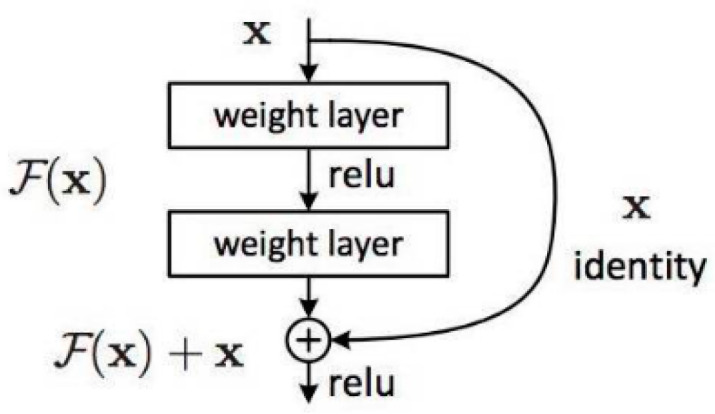
Overview of the residual network.

Resnet is a convolutional neural network proposed by Kaiming He from Microsoft Research, alongside three other people. It won the prize for image classification and object recognition as part of the ImageNet Large Scale Visual Recognition Challenge (ILSVRC) in 2015 [25]. The characteristics of this residual network include the fact that it is easy to optimize and the fact that it can increase the accuracy of recognition with the addition of considerable depth. The internal residual block uses jump connections, which alleviate the problem of the vanishing gradient caused by increasing depth in a deep neural network. An overview of the residual network is shown in Figure 2.

#### 2.3.2. Xception

Xception [26] is another Google modification of Inception-V3 [27]. The author of [26] believes that the correlation between channels and the spatial correlation should be handled separately. Therefore, depthwise separable convolution is also used to replace the convolution operation in Inception-v3, which can improve the effect of the model with only a small increase in network complexity.

#### 2.3.3. MobileNet

In 2012, AlexNet won the first prize in the ImageNet competition [28]. Since then, even deeper neural networks have been proposed, such as the excellent VGG series, GoogleNet, and the Resnet series. However, as these networks become deeper, the huge storage pressure and computing overheads caused by model computation begin to seriously affect the application of deep neural networks in some low-power fields, such as Resnet152 in the Resnet series, where the network layer reached 152 layers and the weight of the model reached hundreds of megabytes.

MobileNet is a network architecture that was released by Google in 2017. It is a convolutional neural network with a small model volume, fewer training parameters, and fewer requirements for computation [29]. Aimed at making full use of limited computing resources and maximizing the accuracy of the model, it is one of the models most commonly deployed in edge computing. The main innovation of MobileNet is the replacement of regular convolution with depthwise separable convolution and the use of a width multiplier to reduce the number of parameters. In addition, it can achieve better data throughput with only a minimal cost to its precision.

The performance of the abovementioned three networks on Pascal VOC 2012 is shown in Table 1.

## 3. Materials and Methods

### 3.1. Dataset

The pictures used in this study are from the Sichuan Wangjiang Farming Muli Base. The dataset was captured by the surveillance camera in the base, with a resolution of 1920 × 1080 pixels, which was located on the upper side of the pig house, shooting at about 45 degrees from top to bottom. The videos were taken from October to November 2020. Frames were extracted based on 2 s intervals. Most of the images in the dataset are clear, though some pigs in a few images were blurry when static images were captured due to movement. In this study, such images were also added to the dataset in order to increase its robustness. The dataset marked the position of each pig whose posture could be clearly judged, as well as the positions of any pigs visible at the edges of the frame, and marked four common postures, including standing, lying on the stomach, lying on the side, and exploring. Finally, this article collected 1550 multi-target object detection images, and further segmented the datasets into the standing, lying on the side, lying on the side, and exploring categories, leading to a total of 3200 images in the detection results. The detection and classification datasets were divided into training and testing sets at a ratio of 7:3.

### 3.2. Image Pre-Processing

#### 3.2.1. Single-Target Extraction by Target Detection

**Figure 3:**
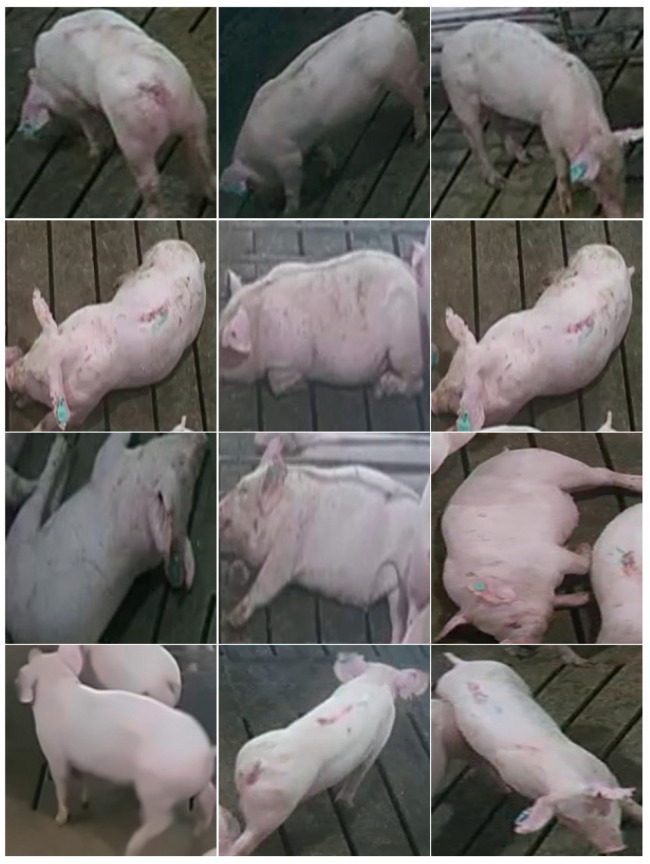
A single pig photo collection extracted by YOLOV5.

This paper used the YOLOv5 network to extract a single target from the input multi-pig images. There are four versions of the object detection network given by YOLOV5, which are YOLOv5s, YOLOv5m, YOLOv5l, and YOLOv5x. The goal of this step was to detect a single pig body as quickly as possible. We tested YOLOv5 on the constructed dataset. YOLOv5s showed the fastest speed and relatively high accuracy, so we chose it as the model for this step. The multi-angle single-pig image size was adjusted to 608 × 608 through adaptive image scaling, and it was sent to the network. The original 608 × 608 × 3 image was input into the newly added focus structure; first, it was turned into a feature map of 304 × 304 × 12 by using the slicing operation, and then, it became a feature map of 304 × 304 × 32 after a convolution operation with 32 convolution kernels. After the input into the structure of feature pyramid networks (FPN) combined with the path aggregation network (PAN), GIoU_Loss was used as the loss function of the bounding box frame to perform the final target detection. The recognition result is shown in Figure 3.

#### 3.2.2. Noise Reduction

In order to enhance the effectiveness of the feature extraction and the accuracy of algorithm recognition, image pre-processing was performed on single-target pictures before semantic-segmentation training. In order to solve the problem of jaggy edges in the test set for semantic segmentation, non-local means (NL-means) filtering [30] was used to denoise the image. The algorithm used redundant information commonly found in natural images to remove noise. Differently from the commonly used bilinear filtering and median filtering [31], which use the local information of the image to filter any noise present, NL-means denoised the entire image, finding similar areas in the image in units of image blocks and then averaging these regions to remove the Gaussian noise in the image more successfully. Color images were first converted to the CIELAB color space, and then, we denoised the L and AB components separately. The formula is as follows:

(1) $$\tilde{u}\left(x\right)=\underset{y\in \Omega_{z}}{\sum }\omega \left(x,y\right)v\left(y\right)$$

$$\omega \left(x,y\right)>0\;and\;\underset{y\in \Omega_{z}}{\sum }\omega \left(x,y\right)=1,\;\forall x\in \Omega ,y\in \Omega_{x}$$

where ω(x,y) is a weight that represents the similarity between pixels *x* and *y* in the original image *v*. The weight is greater than 0, and the sum of the weights is 1. Ω*x* is the neighborhood of pixel *x*.

The method most commonly used to measure the similarity of two image blocks is calculating the Euclidean distance between them [32]. The formula is as follows:

(2) $$\omega \left(x,y\right)=\frac{1}{n\left(x\right)}exp\left(\frac{||\Omega \left(x\right)-\Omega {\left(y)|\right|}_{2,a}^{2}}{h^{2}}\right)$$

where n(x) is a normalized factor, which is the sum of all the weights. After dividing each weight by this factor, the condition of the sum of the weights being 1 is met. h, which is >0, is the filter coefficient, which controls the attenuation of the exponential function to change the weight of the Euclidean distance. Ω(x) and Ω(y) represent the neighborhoods of pixels *x* and *y*, and these neighborhoods are called patch neighborhoods. The block neighborhood is generally smaller than the search area. $||\Omega \left(x\right)-\Omega {\left(y)|\right|}_{2,a}^{2}$ is the Gaussian-weighted Euclidean distance between two neighborhoods, where a, which is >0, is the standard deviation of the Gaussian kernel.

**Figure 4:**
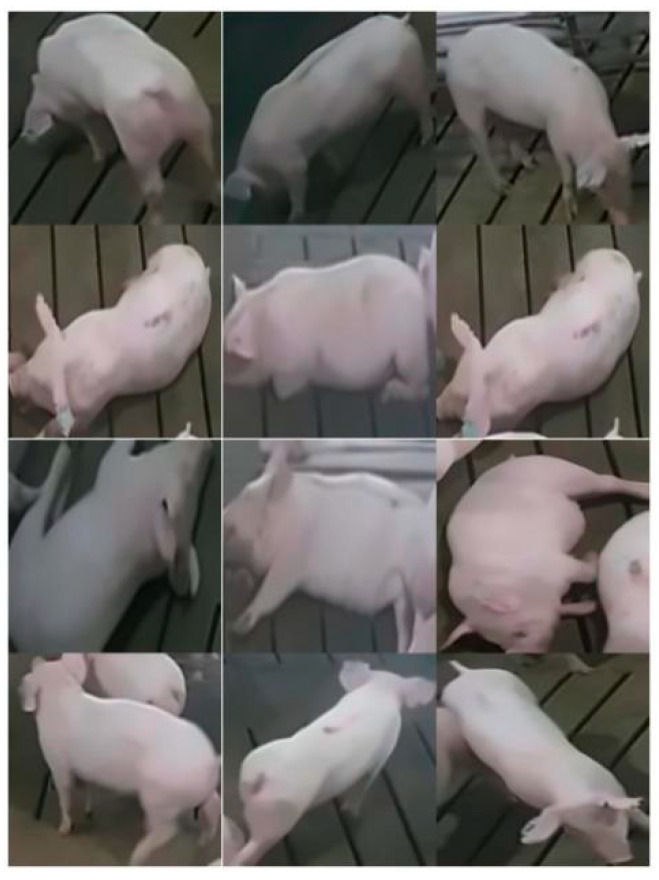
The image after noise reduction.

The image after noise reduction is shown in Figure 4.

### 3.3. DeepLabv3+ Network Structure

**Figure 5:**
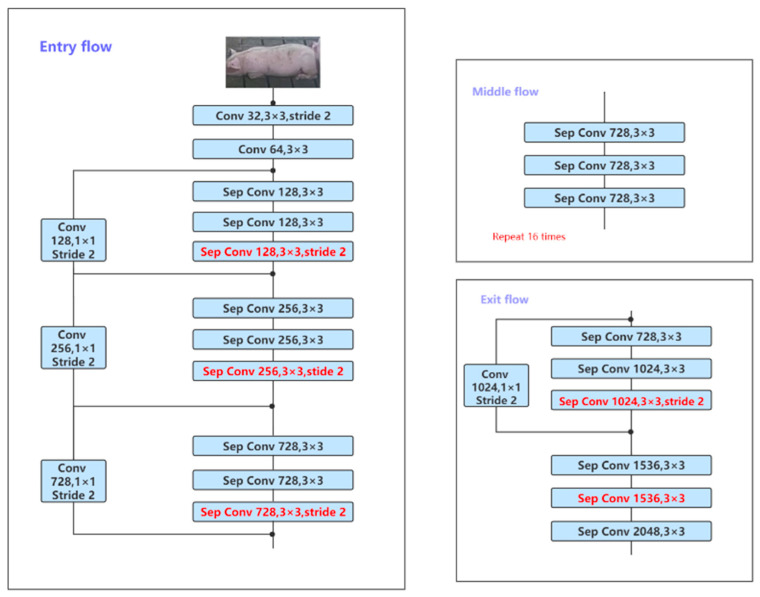
DeepLabv3+’s network structure diagram, which includes three parts: entry flow, middle flow, and exit flow.

In DeepLab v3+, Liang-Chieh Chen et al. used an encoder–decoder to perform multi-scale information fusion while retaining the original dilated convolutions and ASPP layer. Its backbone network uses the Xception model, which improves the robustness and operating speed of semantic segmentation [23]. DeepLabv3+’s network structure is shown in Figure 5. It achieved a mean intersection over union (MIoU) of 89.0% on Pascal VOC, and an MIoU of 82.1% on Cityscape. The greatest improvement of DeepLab v3+ is to regard the DCNN part of DeepLab as an encoder, and up-sample the feature map output by DCNN into the part of the original image size regarded as a decoder, which constitutes the encoder–decoder system. Bilinear interpolation up-sampling is a simple decoder, and the enhancement of the decoder can make the model as a whole obtain good results for the edge features during semantic image segmentation. By experimenting with DeepLab v3 and DeepLab v3+, this study discovered that, when the original DeepLab v3 uses the backbone Resnet-101, the feature map of the subsequent nine layers becomes larger, the amount of calculation greatly increases, and dealing with high-resolution images is time consuming [23]. On this basis, DeepLab v3+ uses modified aligned Xception’s improved Resnet-101, and uses 1 × 1 convolution to reduce the number of channels from low-level features. By inputting the noise-reduced pig pictures into the network where Resnet-101, MobileNet, and Xception are the backbone, after experimental feedback, Resnet-101 shows the best effect. Xception first finds the correlation across a 2D space and then finds the correlation across a 1D space. According to the experimental results, it is believed that, in this experiment, part of the feature information of the pig body was lost, when comparing with the full mapping method’s results.

### 3.4. Experimental Environment

The experimental environment was based on Ubuntu 18.04; the hardware environment was an Intel Xeon E5-2678 48 Core CPU, an NVIDIA TITAN Xp graphics card, and 64 GB of RAM; the programming development environment was CUDA-Toolkit 10.0; the programming language was Python 3.6; the deep-learning framework was Pytorch; and the compiler adopted GCC 4.8.

### 3.5. Experimental Evaluation Index

This article used semantic segmentation accuracy, classification accuracy, MIoU, frequency weighted intersection over union (FWIoU), and loss as evaluation criteria. The definition of the experimental evaluation index is shown in Table 2.

$P_{ii}$ indicates that the original category *i* is predicted as category *i*; $P_{ij}$ indicates that the original category *i* is predicted to be category *j*. The results are divided into the following four situations:

(1) True positive (TP): the prediction result is positive, and the prediction is correct.
(2) True negative (TN): the prediction result is negative, and the prediction is correct.
(3) False positive (FP): the prediction result is positive, but the prediction is wrong.
(4) False negative (FN): the prediction result is negative, but the prediction is wrong.

Acc is used to calculate the ratio between the number of correctly classified pixels and the total number of pixels:

(3) $$\mathrm{Acc}=\frac{\mathrm{TP}+\mathrm{TN}}{\mathrm{TP}+\mathrm{TN}+\mathrm{FP}+\mathrm{FN}}\;\mathrm{Acc}=\frac{\sum_{i=0}^{k}p_{ii}}{\sum_{i=0}^{k}\sum_{j=0}^{k}p_{ij}}$$

The mean intersection over union (MIoU) is a standard measure of semantic segmentation that is used to calculate the ratio of the intersection and the union of two sets of true and predicted values:

(4) $$\mathrm{MIoU}=\frac{\mathrm{TP}}{\mathrm{FP}+\mathrm{FN}+\mathrm{TP}}\;\mathrm{MIoU}=\frac{1}{n+1}\overset{n}{\underset{i=0}{\sum }}\frac{p_{ii}}{\sum_{j=0}^{n}p_{ij}+\sum_{j=0}^{n}p_{ji}-p_{ii}}$$

The frequency weighted intersection over union (FWIoU) sets the weight according to the frequency of each category, and the weight is multiplied by the IoU of each category and summed. In the confusion matrix calculation, the true number of each category is TP+FN, and the total is TP + FP + TN + FN. The calculation formula for the product of the weight of each category and its IoU is as follows, and then, the sum of all the categories is calculated:

(5) $$\mathrm{FWIoU}=\left[\left(\mathrm{TP}+\mathrm{FN}\right)/\left(\mathrm{TP}+\mathrm{FP}+\mathrm{TN}+\mathrm{FN}\right)\right]\times \left[\mathrm{TP}/\left(\mathrm{TP}+\mathrm{FP}+\mathrm{FN}\right)\right]\;\mathrm{FWIoU}=\frac{1}{\sum_{i=0}^{k}\sum_{j=0}^{k}p_{ij}}\overset{k}{\underset{i=0}{\sum }}\frac{\sum_{j=0}^{k}p_{ij}p_{ii}}{\sum_{j=0}^{k}p_{ij}+\sum_{j=0}^{k}p_{ji}-p_{ii}}$$

## 4. Results

### 4.1. Model-Training Method

This study analyzed a classic model based on previous experience gained from many experiments. In DeepLab training, the weights are initialized with the pre-trained weights of Resnet-101 and Xception, respectively, the output step is 16, the loss function uses cross-entropy loss (ce), the batch size is 4, the Lr is 0.01, the poly learning rate drops, the momentum is 0.9, and the decay is 0.0005.

### 4.2. Experimental Comparative Analysis

**Figure 6:**
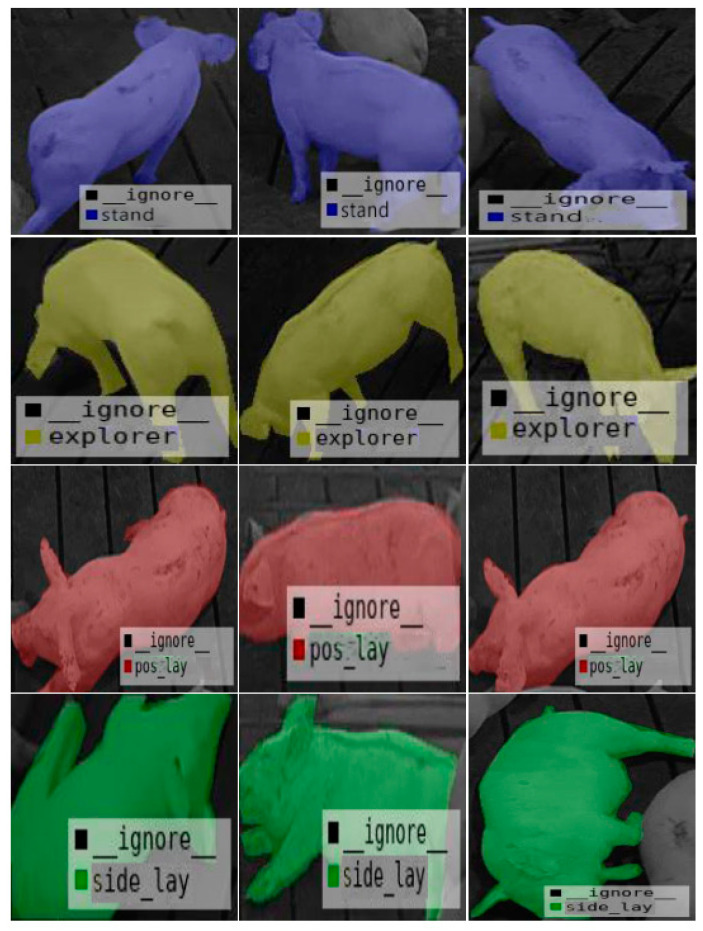
Image after semantic segmentation.

The results of the semantic segmentation are shown in Figure 6. The figure shows the training process and experimental results using the dataset constructed in this paper. The highest semantic-segmentation accuracy rate based on Resnet-101 was 92.45%, and the classification accuracy rate under the four posture types was 92.26%.

**Figure 7:**
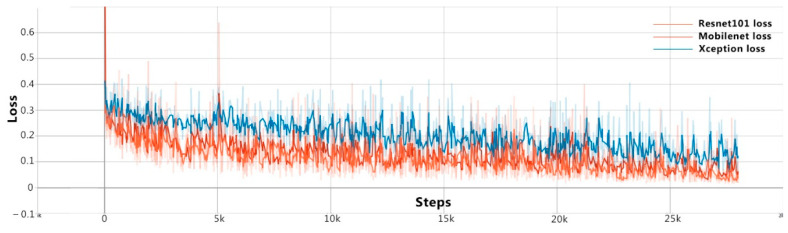
The change in loss for DeepLab v3+ according to the training process.

**Figure 8:**
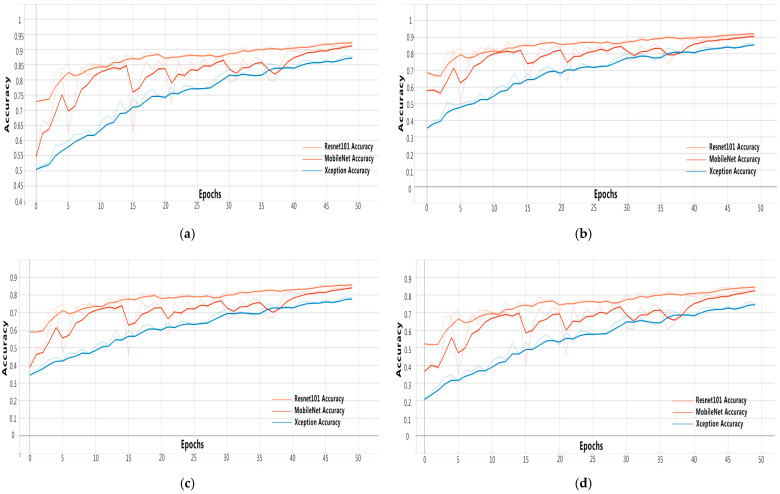
The algorithm training progress: (**a**) semantic-segmentation accuracy of the test set; (**b**) test-set-classification accuracy; (**c**) frequency weighted intersection over union (FWIoU) of the test set; (**d**) mean intersection over union (MIoU) of the test set.

In the process of model training, overall loss changes are shown in Figure 7. Four indicators are mainly used to measure the availability of the model, and the test data of each indicator is shown in Figure 8.

Based on the observation of the figures below, the following conclusions can be drawn:

1. Through the above experimental process, for the feature extraction models Resnet-101, Xception, and MobileNet, there were oscillations in the early training process, but the convergence effect was good in the later period.
2. Resnet had the best classification effect on this dataset, with a classification accuracy of up to 92.26%.
3. Although MobileNet had an absolute advantage in terms of its training speed, and its accuracy in the later training period was close to that of the Resnet training, the accuracy curve fluctuated significantly in the training process and lacked stability.
4. Based on the dataset used in this paper, the Resnet training lasted 7 h and 30 min, the Xception training lasted 7 h and 23 min, and the MobileNet training lasted 1 h and 50 min.

We respectively used three different Backbones for testing. Through comparison, we finally adopted ResNet as our backbone. The detailed test results are shown in Table 3.

### 4.3. Discussion

This study explored an automatic method for recognizing the posture states of pigs on a farm. We could extract key frames from the monitoring image, detect a single pig from a single image in each frame, extract the contours of each pig, and distinguish their posture through the classifier we designed, to determine the effect of posture monitoring in the pig herd. With regard to the feasibility of this method, we must consider the following:

(1) In terms of the recognition accuracy, since our current experimental data source was based on a single camera to the side of the pigs, the observation angle of the pig posture was singular, and the identification of postures by cameras in front or to the side of moving pigs had a certain influence. Therefore, in practical applications, a pig farm should install multiple cameras to collect data in all directions in the environment and assign different weights to calculate pig postures based on different angles.
(2) In terms of the processing speed, in order to realize the real-time monitoring of pig postures, data could be extracted from one frame every 2 s in practical applications. In terms of the computational speed of the model, both YOLOv5 and DeepLab v3+ have good processing speeds, as explained in Section 2.1 and Section 2.3.
(3) This method also has certain limitations. For example, after extracting frames from a video to obtain a static image, it is impossible to determine whether a pig is moving. In the future, video recognition or a recurrent neural network (RNN) could be used to generate a sequence of pictures with which to classify behaviors in order to solve this problem.

Jiazhen Han sought to solve the problem of multi-target recognition [12], while Dan Li proposed that the establishment of a standard dataset for pigs is necessary and would have far-reaching significance [33]. Unlike in other studies, the target of this work was a group of pigs, and this study created the world’s first human-annotated pig-posture-recognition dataset. After an improved combined training method involving target detection and semantic segmentation, this study obtained more accurate recognition than related research and solved the problems related to the difficulty of removing background factors and multi-target recognition.

Based on the above discussion, we believe that the method proposed in this paper can effectively identify pig postures and could be used as the basis for an automated breeding system.

## 5. Conclusions

This study applied deep-learning technology for pig-posture recognition and proposed a joint training method involving target detection and semantic segmentation. First, a dataset containing images of multiple pigs was collected; then, target detection was performed using the collected images to extract individual pigs. These pigs were then classified and labeled by professionals. To the best of our knowledge, this is the world’s first dataset of pig postures. After the construction of the dataset was complete, in order to solve the problem of edge aliasing in semantic segmentation, non-local average filtering was used for image pre-processing. After the dataset was formed, the improved DeepLab v3+ was used to conduct multiple experiments using the Resnet, Xception, and MobileNet networks. The experimental results show that the accuracy of the semantic segmentation reached 92.45%. Resnet had the best classification performance, and its classification accuracy reached 92.26%. Therefore, the accuracy of the pig-posture extraction and classification method developed in this study meets the requirements for practical applications. It may have certain value in practical applications of pig-posture assessments on pig farms, and also provides new ideas regarding posture identification among related animals.

## Figures and Tables

**Table 1:** The classification network structures mentioned in this paper. In this study, a total of three classical classification networks were used. Performance of each network on Pascal VOC 2012 is presented.

Backbone	MIoU in Val
Resnet	78.43%
MobileNet	70.81%
Xception	/

**Table 2:** Definition of the experimental evaluation index.

		Expected Results	
		Positive	Negative
**Actual Results**	Positive	TP	FP
	Negative	FN	TN

**Table 3:** The semantic-segmentation accuracy and classification accuracy of each epoch, based on three experimental methods.

	Category	Epoch10	Epoch20	Epoch30	Epoch40	Epoch50
Resnet	Acc	84.85	85.16	89.72	90.58	92.45
	AccClass	82.22	83.8	88.37	89.67	92.26
Xception	Acc	66.1	73.7	83.7	83.66	87.53
	AccClass	57.99	66.4	79.98	80.19	85.68
MobileNet	Acc	84.86	83.58	79.04	89.6	91.69
	AccClass	82.82	83.53	77.14	88.62	91.03
